# Poisson–Gaussian Noise Analysis and Estimation for Low-Dose X-ray Images in the NSCT Domain

**DOI:** 10.3390/s18041019

**Published:** 2018-03-29

**Authors:** Sangyoon Lee, Min Seok Lee, Moon Gi Kang

**Affiliations:** School of Electrical and Electronics Engineering, Yonsei University, 50 Yonsei-Ro, Seodaemun-Gu, Seoul 03722, Korea; sangyoonlee@yonsei.ac.kr (S.L.); gasinamul@empal.com (M.S.L.)

**Keywords:** low-dose X-ray, non-subsampled contourlet transform (NSCT), Poisson–Gaussian noise, noise analysis, noise estimation

## Abstract

The noise distribution of images obtained by X-ray sensors in low-dosage situations can be analyzed using the Poisson and Gaussian mixture model. Multiscale conversion is one of the most popular noise reduction methods used in recent years. Estimation of the noise distribution of each subband in the multiscale domain is the most important factor in performing noise reduction, with non-subsampled contourlet transform (NSCT) representing an effective method for scale and direction decomposition. In this study, we use artificially generated noise to analyze and estimate the Poisson–Gaussian noise of low-dose X-ray images in the NSCT domain. The noise distribution of the subband coefficients is analyzed using the noiseless low-band coefficients and the variance of the noisy subband coefficients. The noise-after-transform also follows a Poisson–Gaussian distribution, and the relationship between the noise parameters of the subband and the full-band image is identified. We then analyze noise of actual images to validate the theoretical analysis. Comparison of the proposed noise estimation method with an existing noise reduction method confirms that the proposed method outperforms traditional methods.

## 1. Introduction

Since their discovery by Röntgen, X-rays have been the subject of research in a broad variety of fields. One of the first uses of X-rays was in the medical field to obtain information on the interior of the body without dissection. In addition to the basic technique of X-ray imaging of still-cuts, a number of imaging systems have been developed, including methods for using moving X-ray images to observe the cardio-vascular system and 3D imaging through computed tomography. An X-ray beam is partially absorbed by structures within the body in a process known as attenuation, and the detector on the other side of the body absorbs this attenuated X-ray to produce an X-ray image. The high permeability of X-rays owing to their high energies can have negative effects on the human body. It is also continuously concentrated, such as lead and mercury, and has a detrimental effect. Although risk from medical radiation continues to decrease with the application of isotopes with shorter half-lives, even very small dosages can be dangerous under frequent exposure. To reduce the risk of exposure, methods for using low-dose X-rays have been developed in recent years. In low-dose X-rays, the incident photon density and the photon unevenness are reduced, resulting in a much higher quantum noise concentration relative to the anatomical information of the human body, which in turn degrades image quality. Many of the existing algorithms for X-ray image noise reduction algorithms assume a Poisson distribution of quantum noise [1]; however, Elbakri and Fessler [2] demonstrated that the actual noise combines Poisson and Gaussian distribution, with the Poisson distribution arising from quantum noise and the Gaussian distribution arising from thermal noise generated by sensors and other electronic devices.

Because Poisson noise is signal-dependent, it is difficult to design a general noise reduction algorithm; to address this, variance stabilization transforms (VSTs) have been introduced. The Anscombe transform targeting the Poisson noise was proposed by Anscombe in [3] and extended to the generalized Anscombe transform (GAT) and inverse transform for Poisson–Gaussian noise by Makitalo and Foi in [4]. Using this VST, Poisson–Gaussian noise is converted into Gaussian noise with constant noise variance, enabling the effective application of a number of noise reduction algorithms based on Gaussian noise [5,6,7,8,9,10,11]. Other methods for solving Poisson–Gaussian noise directly without using variance stabilization have also been developed. A noise-reduction method based on total variation regularization under a new model for Poisson distribution was proposed in [12], while the use of stochastic distance, a non-local measure based on symmetric divergence, instead of similarity measure, was proposed in [13]. A Poisson noise reduction method based on the Poisson PCA was proposed in [14]. Another effective method for reducing Poisson noise is to use a multiscale transform, the most commonly used of which is [15]. The Expectation–Maximization (EM) algorithm is used for image restoration based on the penalized likelihoods formulized in the wavelet domain [16]. Poisson–Gaussian unbiased risk estimate (PURE), a noise reduction method based on the extension of Stein’s unbiased risk estimate (SURE) in Poisson–Gaussian conduction is also performed in the wavelet domain [17].

Although the wavelet transform is an efficient computational algorithm that performs well in image noise reduction, it has fundamental drawbacks, including immobility, directional invariance, and directional selectivity. In addition, small coefficients are likely to arise from noise, while large coefficients are more likely to be caused by important signal characteristics. Discrete wavelet transforms (DWTs) consist of vertical and horizontal filters and cannot not be expressed in other directions. Therefore, they can only find discontinuities of straight lines at the edges, but cannot represent smoothness along the contours [18]. Furthermore, conventional wavelet transforms are less efficient for line- and curve-specific (edge) analysis in the two-dimensional domain [19]. Thus, a typical wavelet transform does not have optimal properties for the analysis of two-dimensional signals such as natural images. To overcome these drawbacks, multiscale and directional representations have recently been proposed. Ridgelet [20], curvelet [21], and contourlet [18,22,23] methods have been introduced to efficiently solve the 2D multiscale and directionality problems. The primary characteristics of contourlet transform (CT) are multi-resolution, localization, directionality, and anisotropy; in particular, the directionality property overcomes the well-known limitations of the commonly used separable transforms by enabling the resolution of unique directional features characterizing an analyzed image. Although CT can display the contours and textures of an image very sparsely, because up- and down-sampling is included in the CT process, it does not produce a one-to-one correspondence between coarser and finer levels. In addition, CT has a shift-variance property, with produced values changing according to sub-sampling position. To solve this problem, a non-subsampled contourlet transform (NSCT) was proposed in [24]. Unlike CT, NSCT does not use up- and down-sampling, giving it a shift-invariant property and a one-to-one correspondence between pixel positions at different levels.

The contribution of this paper is twofold. First, we analyze the Poisson–Gaussian noise for low-dose X-ray images in the NSCT domain, and show that it also has a Poisson–Gaussian distribution. This is done by analyzing the noise distributions of simulated noisy images and the noiseless original image in the non-subsampled pyramid (NSP) domain and then analyzing the Poisson–Gaussian noise distribution in non-subsampled directional filter banks (NSDFBs). The Poisson–Gaussian noise in the NSCT domain is then analyzed by linking these analyses. To confirm the consistency of this theoretical analysis with the actual results, we then analyze the noise distribution of the actual low-dose X-ray images in the NSCT domain. Second, we estimate the Poisson–Gaussian noise in each subband. Noise estimation is the most important component in noise reduction processing and has the most impact on results; correspondingly, we estimate the Poisson–Gaussian noise in NSP, NSDFB, and NSCT domains using the analyzed noise distribution. We observe the relationship between the noise parameters of the full-band image and the noise parameters of the NSP subband and then analyze the relationship between the noise parameters of the NSP subband and those of the NSCT subband after passing through the NSDFB. Finally, the advantages of the proposed method relative to the conventional method are demonstrated by applying the estimated noise parameter to an existing noise reduction method.

The paper is organized as follows. The Poisson–Gaussian noise model and the NSCT are described briefly in Section 2. In Section 3, we analyze the Poisson–Gaussian noise in the NSP, NSDFB, and NSCT domains and use the results to estimate the noise parameters in each decomposition domain in Section 4. The results of noise reduction using proposed noise analysis and estimation method are compared with those obtained from a conventional method in Section 5 and, finally, Section 6 presents our conclusions and future work.

## 2. Preliminaries

### 2.1. Poisson–Gaussian Noise Model

The target images for noise analysis in this work are low-dose X-ray images. X-ray images are known to have signal-dependent Poisson noise; however, noise added by the sensor itself and other electronic devices during measurement results in noise with a distribution following a Poisson–Gaussian mixture model. The generic signal dependent noise model in [25] is given by

(1) $$y=x+\eta \left(x\right)\delta ,$$

where *y* is the acquired image, *x* is the original image, $\eta \left(x\right)$ is the standard deviation of the noise distribution, and δ is the independent Gaussian noise with zero-mean and a standard deviation equal to one. Because of the signal-dependent noise, the standard deviation of the noise η is a function of the original signal, *x*. In a Poisson–Gaussian image noise model, the noise variance $\eta {}^{2}\left(x\right)$ can be separated into signal-dependent and signal-independent components (Poisson and Gaussian noise, respectively). The variance of the noise term can thus be expressed as

(2) $$\eta {}^{2}\left(x\right)=\alpha x+\sigma^{2},$$

where α and σ are the Poisson noise parameter and the standard deviation of the Gaussian noise, respectively. The signal-dependent component of the noise term is proportional to the original image, while the signal-independent component is an additive constant term.

**Figure 1:**
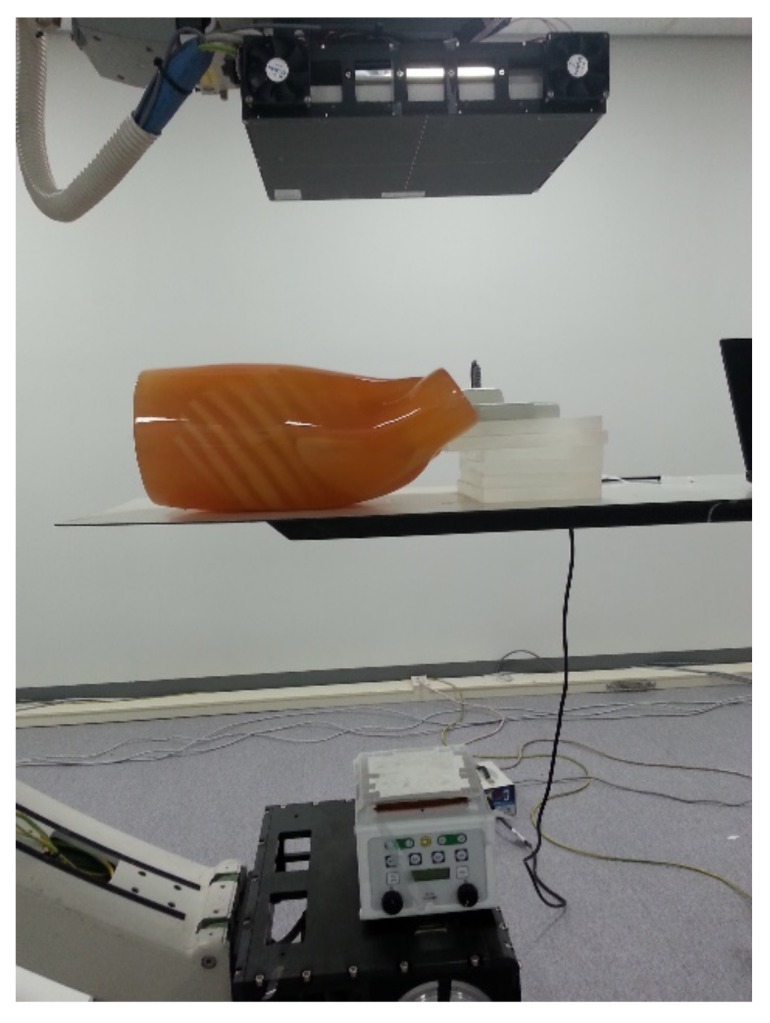
A clinical angiography prototype system and chest phantom (Multipurpose Chest Phantom N1 “LUNGMAN”, Kyoto Kagaku, Kyoto, Japan).

**Figure 2:**
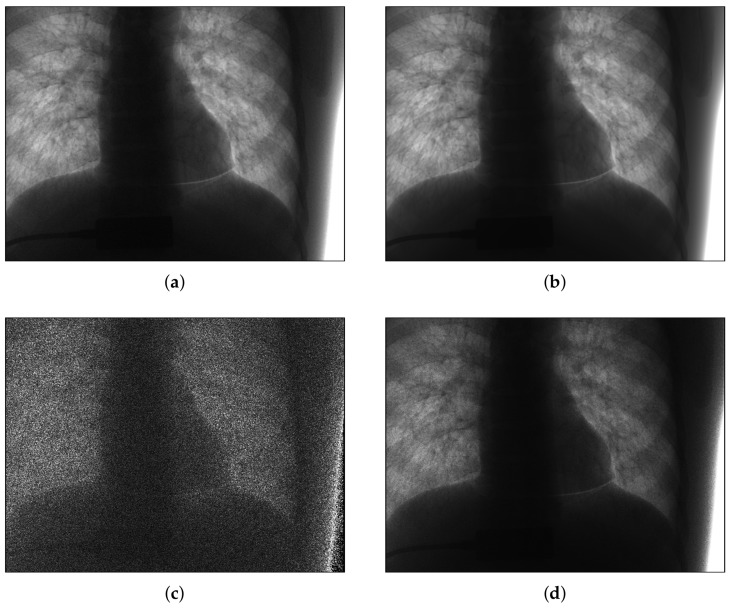
Noise analysis of actual image. (**a**) observed image; (**b**) noiseless image (average of 100 noisy images); (**c**) noise image (difference between observed and noiseless images); (**d**) variance of noise images.

To characterize the noise patterns in low-dose X-ray images, we analyzed the noise in 100 captured images. The image data for the evaluation were acquired using a clinical angiography prototype system supported by Samsung Electronics and a chest phantom (Multipurpose Chest Phantom N1 “LUNGMAN”, Kyoto Kagaku, Kyoto, Japan), yielding life-like radiographs very close to actual clinical images, as shown in Figure 1. The size of each digital image was 1024 × 768 pixels, while the image intensity had 12-bit precision. The image acquisition environment was as follows. The source to image-receptor distance (SID) was 120 cm, and the source to object distance (SOD) was 70 cm. The radiation exposure level was 1.94 μGy/pulse and the scan parameters were 62 kVp and 40 mA. The average of the images is shown in Figure 2b. Since 100 images are averaged, the variance of the noise is reduced to 1/100. The maximum value of the noise variance in Figure 2d was about 1820. Then, in the averaged image, the strongest noise had a variance of about 18.2, and the standard deviation of about 4.266. Because the δ of Equation (1) is a normal distribution, 99.7% of the noise is in the range of $-3\eta \left(x\right)$ to $3\eta \left(x\right)$. Using the maximum standard deviation of the noise of the averaged image obtained above, 99.7% of the noise in the averaged image was within the range of −12.8 to 12.8. Since the precision of the input image was 12-bit, it had a pixel value of 0 to 4095, and most of the noise in the averaged image had the range of about 0.3% of the pixel value range. Averaging over 100 images did not completely eliminate noise, however, since the range of noise was negligible compared to the range of pixel values, we assumed that the averaged image with maximum variance of Poisson–Gaussian noise lower than 20 to be noiseless image. Using the differences between the acquired images and this assumed original image, noise-only images (Figure 2c) were calculated and their variance (Figure 2d) was used to analyze the noise. Figure 2c,d show that the noise level is strong in places where the intensity of the original image is bright and weak in a dark places, suggesting that the noise in low-dose X-ray images depends on the signal.

**Figure 3:**
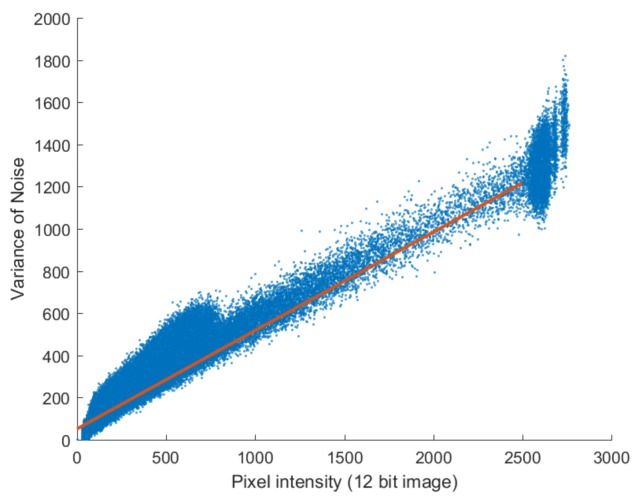
Noise variance against noiseless pixel intensity estimated from noisy images. The slope of the solid line is the Poisson noise parameter, α, and the *y*-axis intercept is the variance of the Gaussian noise, $\sigma {}^{2}$.

We then plotted the pixel values of the noiseless image (Figure 2b) against the variance of noise images (Figure 2d) in Figure 3 and found a linear relationship between the intensity of the noiseless pixels and the variance of the noise. The solid line in Figure 3 was drawn by putting the noise parameters estimated in Section 4 into Equation (2). As shown in Figure 3, the variance of the noise according to the pixel intensity corresponds with Equation (2); the slope can be interpreted as the parameter of the Poisson noise (α), while the *y*-axis intercept can be interpreted as the variance of the Gaussian noise ($\sigma {}^{2}$).

### 2.2. Non-Subsampled Contourlet Transform

**Figure 4:**
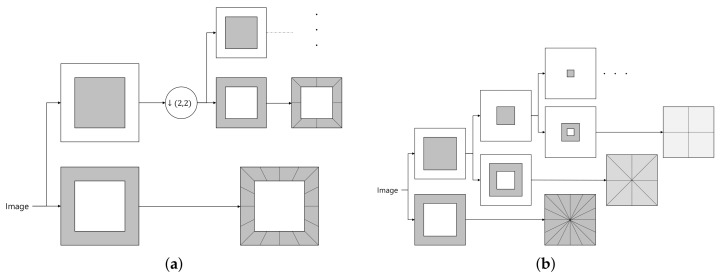
CT and NSCT decomposition schemes. (**a**) CT; (**b**) NSCT.

CT [18] is a transform that separates the subbands by band-pass frequency and direction using a Laplacian pyramid (LP) [26] and directional filter bank (DFB) [27]. In the CT process, as shown in Figure 4a, LP decomposition is first used to separate an image into a low-pass subband and band-pass subbands and then a DFB is applied to the band-pass subbands to separate the subband region of each direction. The LP separates the images by frequency band and the DFB separates images by direction rather than by the frequency band. The filter of the LP is used to separate high-frequency and low-frequency regions, and then low-frequency regions are repeatedly separated into high-frequency and low-frequency regions. In this manner, images are separated by scale level. The DFB is efficiently implemented through *N*-level tree structure decomposition. In each tree structure decomposition, an hourglass filter and a fan filter are applied to divide the image into two parts according to directions. Through the decomposition of *N* iterative tree structures, we divide into $2^{N}$ wedge-shaped frequency bands. The advantage of the DFB is directional selectivity and efficient structure. Since the directionality is considered by using the DFB, the CT can complement the disadvantage of wavelet transform and express the contour of the image. A wavelet transform stronger in the vertical or horizontal patterns corresponds to a stronger contourlet transform in the curves or contours, respectively. LP and DFB both include subsampling procedures. Therefore, CT also performs subsampling, which means that the size of the subband after CT is different from that of the original image. This causes the transform to have a shift-variant property, which causes major issues in noise reduction. To solve this problem, Da Cunha et al. proposed the non-subsampled contourlet transform (NSCT) in [24], which eliminates the subsampling process in CT. Like CT, NSCT decomposes into scale and direction; unlike CT, it has no sub-sampling process. To achieve shift invariance, NSCT uses a non-subsampled pyramid (NSP) and a non-subsampled directional filter bank (NSDFB) for multiscale and directional decomposition, respectively. NSP and NSDFB are constructed by eliminating the downsamplers and upsamplers in LP and DFB, respectively, and, therefore, NSCT has no downsamplers and upsamplers. The multiscale shift-invariant property of the NSCT is obtained from the shift-invariant filter structure. The NSP can generate a sub-image comprising a low-frequency image and a high-frequency image, each having the same size as the source image. The NSDFB then decomposes the high-pass subband into multiple directional subbands. This scheme is iteratively repeated in the low-pass subband. Figure 4b shows the NSCT decomposition process. As there is no subsampling process, the position of the child node is the same as that of the parent node.

The NSCT decomposition process is described in detail as follows. First, in the NSP decomposition process, a low-pass and a high-pass filter are used to decompose the image. The input image is decomposed into low- and high-frequency bands through low- and high-pass filters, respectively. The separated high-frequency band is used as the detail layer, while the low-frequency band signal is further separated into a lower frequency and a band-pass frequency band by using the low- and high-pass filters, respectively, of the next scale level. If the total scale level at which the input image is divided is *M*, NSP decomposition filtering is repeated *M* times; here, *m* (m=1,2,⋯,M) denotes each scale level, where m=1 representing the finest level and m=M the coarsest level. $G_{0}$ denotes an original image, $G_{m}$ and $L_{m}$ denote, respectively, the low-frequency band and high- or band-pass band at the *m*-th scale level, with 1≤m≤M. In the next step, directional decomposition, the detail layer of the NSP ($L_{m}$) is separated directionally through the NSDFB to form the NSCT subband. The number of directions separated by NSDFB should be an exponential power of two, with the number of directional levels denoted by $N_{m}$ and each directional level denoted by *n* ($n=0,1,\cdots ,N_{m}-1$) and the *n*-th directional subband at the *m*-th scale level denoted by $L_{m,n}$.

## 3. Poisson–Gaussian Noise Analysis in the NSCT Domain

Before using actual images, we analyzed the noise using simulated images. Using high-dose X-ray images assumed to be a noise-free, 100 noisy images were created for noise analysis by generating and adding artificial noise, first in the form of Poisson-contaminated images and then in the form of Poisson–Gaussian-contaminated image. Noise was generated using the imnoise function in MATLAB’s (R2017b, MathWorks, Natick, Massachusetts, USA) image processing toolbox. The noise parameters were set to α=0.0304 for Poisson-contaminated images, and α=0.0844 and $\sigma {}^{2}=32.5125$ for Poisson–Gaussian-contaminated images. To analyze the noise in the NSCT domain, we analyze the images following NSP and NSDFB in a step-by-step fashion.

The noise analysis utilized the low-bands of the noiseless image and the variance of the coefficients of the noisy images. The noisy images were first separated into subbands using a multiscale transform and then the variances of the coefficients of these 100 subbands were calculated at a fixed scale level, direction level, and pixel position. The noiseless image was used in only the low-band in the multiscale transform. The Poisson and Poisson–Gaussian noises were analyzed in terms of the relationship between $\bar{G}$, the low-band coefficient of the noiseless image $\bar{y}$, and Var(L), the variance of decomposed noisy image coefficients.

### 3.1. Poisson Noise Analysis

**Figure 5:**
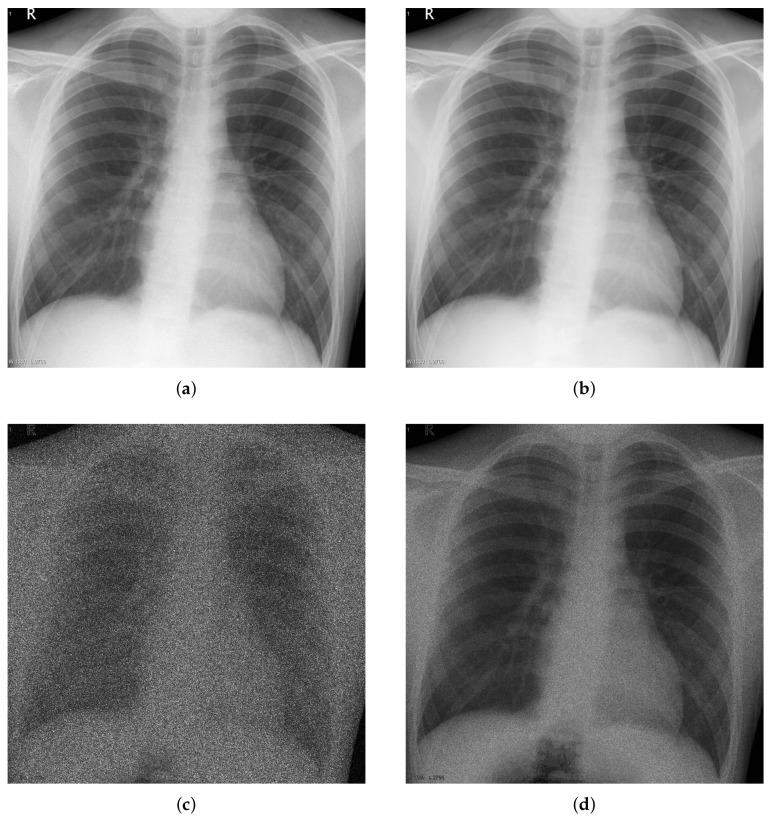
Noise analysis of Poisson noise. (**a**) Poisson noise-added image; (**b**) noiseless image (high-dose X-ray image); (**c**) noise image (artificially generated); (**d**) variance of noise images.

**Figure 6:**
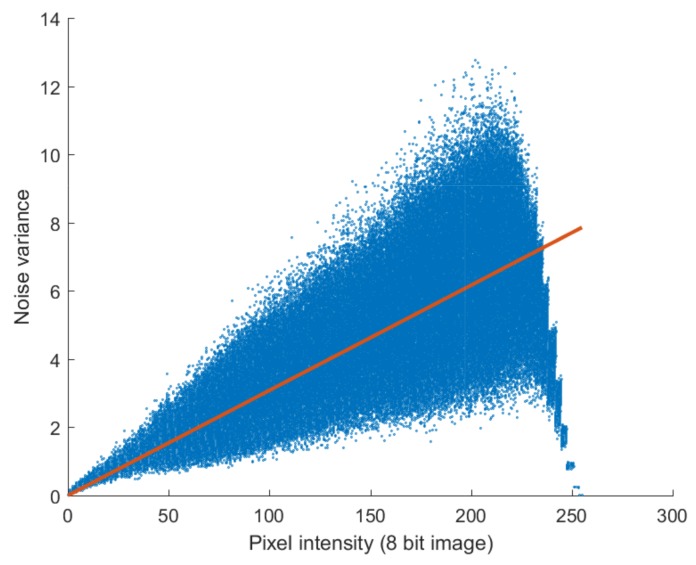
Noise variance against noiseless pixel intensity for Poisson simulated noisy images.

Poisson noise-generated images were analyzed using the methods shown in Figure 2 and Figure 3. Figure 5c,d show that the Poisson noise has signal-dependent tendencies similar to those in actual images, with the noise becoming stronger in bright areas. Figure 6 shows that the linear relationship between noiseless pixel intensity and the variance of the noise images takes the form $\eta {}^{2}\left(x\right)=\alpha x$ because only Poisson noise is present. Near the maximum value of pixel intensity, the noise variance is rather low because the noise is above the maximum value of the image, resulting in saturation.

**Figure 7:**
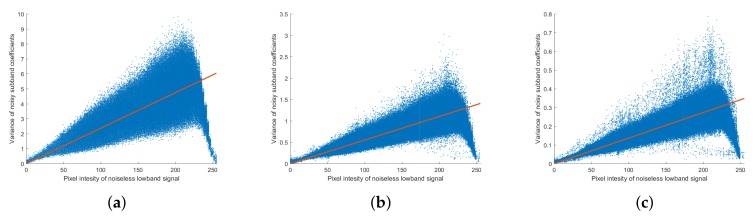
Noise analysis of Poisson noise in the NSP domain. Variance of noise subband coefficient ($\mathrm{Var}(L_{m})$) against pixel intensity of noiseless low-band image (${\bar{G}}_{m}$) at the scale level (**a**) m=1; (**b**) m=2; (**c**) m=3.

We then analyzed the Poisson noise in the NSP domain using the NSP-decomposed low-band layer of the noiseless image (${\bar{G}}_{m}$) and the detail layers of the noisy images ($L_{m}$). The variance of the noise was estimated using the variance of the detail layer coefficients of the noisy images; Figure 7 shows the noise variance of the detail layer as a function of the low-band pixel density distribution of the noiseless image. It is seen that the noise of the detail layer follows the Poisson distribution of the coefficients of the noiseless image low-band layer and that the value of the noise coefficient, α, varies with the scale level, *m*. The noise distribution trends in the NSP domain were the same at all scale levels, and the variance of the noise became smaller as the scale level became coarser. The noise of NSP detail layer can be expressed as

(3) $$\eta {}_{m}^{2}\left(L_{m}\right)=\alpha {}_{m}{\bar{G}}_{m},$$

where $\alpha {}_{m}$ is the Poisson noise parameter at scale level *m* in NSP domain. As *m* increases, the bandwidth of the low-band decreases, which in turn reduces the noise and therefore the value of the Poisson noise parameter, $\alpha {}_{m}$, decreases. This is because the coarser the scale level, the narrower the bandwidth of the NSP low-pass filter; the detailed description is covered in Section 4.

**Figure 8:**
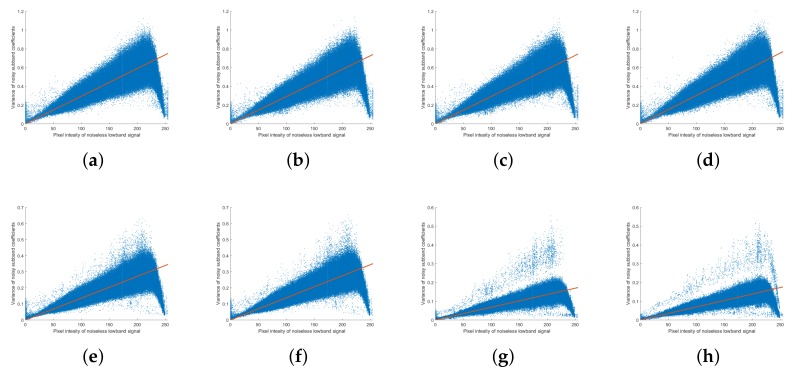
Noise analysis of Poisson noise in the NSCT domain. Variance of noise subband coefficients ($\mathrm{Var}(L_{m,n})$) against pixel intensity of noiseless low-band image (${\bar{G}}_{m}$) for the scale level *m* and direction level *n* (**a**) m=1,n=0; (**b**) m=1,n=1; (**c**) m=1,n=2; (**d**) m=1,n=3; (**e**) m=2,n=0; (**f**) m=2,n=1; (**g**) m=3,n=0; (**h**) m=3,n=1, where $N_{1}=8,N_{2}=4$, and $N_{3}=2$.

The decomposed NSP detail layer was then converted to an NSCT layer through NSDFB. As it has a wider high-pass bandwidth, the finer level was decomposed into more directional levels using $N_{1}=8$, $N_{2}=4$, and $N_{3}=2$. As was done for the NSP domain, we analyzed the relationship between the noiseless low-band pixel intensity, ${\bar{G}}_{m}$, and the noise variance of NSCT coefficients, Var($L_{m,n}$); the results are plotted in Figure 8. The results for scale level m=1 are plotted in Figure 8a–d, scale level m=2 are plotted in Figure 8e,f, and for scale level m=3 are plotted in Figure 8g,h. Similar to the noise distribution in the NSP, the noise distribution for the subband of the noisy image in the NSCT domain follows a Poisson distribution. It is seen that the noise variance after passing through NSDFB is smaller than the noise variance at the NSP detail layer; furthermore, at a given scale level, the distribution of the NSCT noise does not change with directional decomposition. Since the filter of NSDFB has the same energy irrespective of the direction, it is affected only by the number of direction levels to be decomposed and the noise is distributed equally as per that number. This can be expressed as

(4) $$\eta {}_{m,n}^{2}\left(L_{m,n}\right)=\alpha {}_{m,n}{\bar{G}}_{m},$$

where $\alpha {}_{m,n}$ is the Poisson parameter for scale level *m* and directional level *n* in the NSCT domain. Note that $\alpha {}_{m,n}$ has a constant value for a given *m* regardless of *n*.

### 3.2. Poisson–Gaussian Noise Analysis

**Figure 9:**
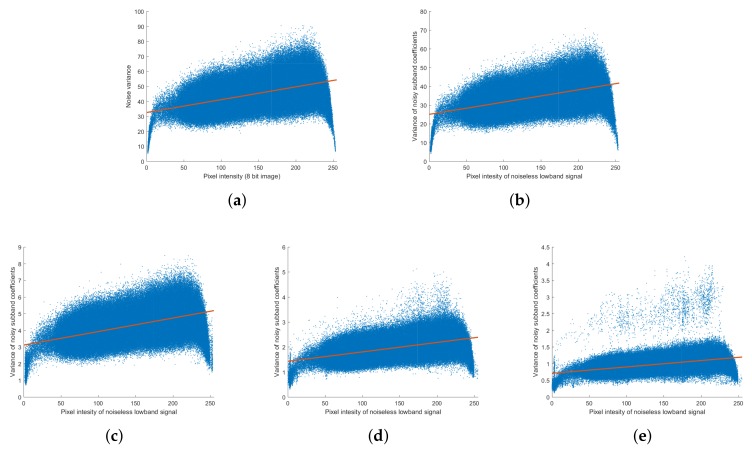
Noise analysis of Poisson–Gaussian noise. (**a**) variance of noise against noiseless pixel intensity; (**b**) variance of noise subband coefficients in NSP domain ($\mathrm{Var}(L_{m})$) against pixel intensity of mean low-band image (${\bar{G}}_{m}$) for m=1; (**c**,**d**,**f**) variance of noise subband coefficients in the NSCT domain ($\mathrm{Var}(L_{m,n})$) against pixel intensity of mean low-band image (${\bar{G}}_{m}$) at scale level *m* and direction level n=0 for (**c**) $m=1,N_{1}=8$; (**d**) $m=2,N_{2}=4$; (**e**) $m=3,N_{3}=2$.

In the succeeding analysis, both Poisson and Gaussian noise were generated and added to an image and the Poisson–Gaussian noise was analyzed using the methodology applied in the analysis of Poisson noise. After analyzing the Poisson–Gaussian noise without multiscale decomposition, the noise in the NSP and NSCT domains were then analyzed in order. The noise variance against noiseless image pixel intensity is shown in Figure 9a. In the full-band image without decomposition, the signal-dependent trend is similar to that in the Poisson noise case but has an added constant; this is similar to the results of the analysis using real images plotted in Figure 3. The variance of the Poisson–Gaussian noise is expressed in the form of Equation (2).

Figure 9b shows the results of the Poisson–Gaussian noise analysis in the NSP domain at scale level m=1. As in the Poisson noise analysis, the values of the noise parameters for each scale level are different, but the trends of noise distributions are all the same; therefore, it is plotted for only one scale level. In the figure, the variance of the noisy detail layers follows a linear relationship with the pixel intensity of the low-band of the noiseless image. The Poisson–Gaussian noise in the NSP domain can be expressed as

(5) $$\eta {}_{m}^{2}\left(L_{m}\right)=\alpha {}_{m}{\bar{G}}_{m}+\sigma {}_{m}^{2},$$

where $\sigma {}_{m}^{2}$ is the variance of Gaussian noise at scale level *m* in NSP domain. Like Poisson noise, the Gaussian noise $\sigma {}_{m}^{2}$ becomes smaller as scale level becomes coarser.

The Poisson–Gaussian noise analysis in the NSCT domain is shown in Figure 9c–e. The figures show the noise analysis for only one direction level at each scale level because the distribution of the noise at a given scale level is unaffected by the direction level. In the NSCT decompositions, the Poisson–Gaussian noise distribution also follows a distribution in which a constant (Gaussian) noise is added to noise with a low-band signal (Poisson) dependence, which is given as follows:

(6) $$\eta {}_{m,n}^{2}\left(L_{m,n}\right)=\alpha {}_{m,n}{\bar{G}}_{m}+\sigma {}_{m,n}^{2}.$$

In conclusion, the Poisson–Gaussian noise comprising a combination of original signal-dependent Poisson noise and independent Gaussian noise has low-band-dependent Poisson–Gaussian distribution even after multiscale conversion. The value of the noise variable is different for each scale level, as is discussed in the next section.

### 3.3. Real Image Noise Analysis

**Figure 10:**
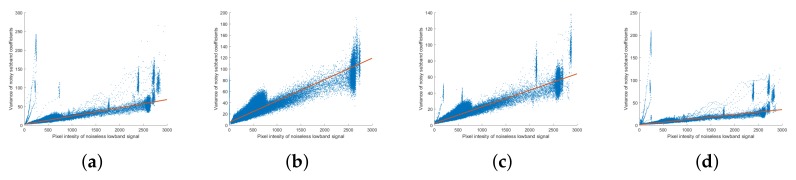
Noise analysis of real image in the NSP and NSCT domains. (**a**) variance of noise subband coefficients ($\mathrm{Var}(L_{m})$) against pixel intensity of mean low-band image (${\bar{G}}_{m}$) at the scale level m=3; (**b**–**d**) variance of noise subband coefficients ($\mathrm{Var}(L_{m,n})$) against pixel intensity of mean low-band image (${\bar{G}}_{m}$) at the scale level *m* and direction level n=0 for (**b**) $m=1,N_{1}=8$; (**c**) $m=2,N_{2}=4$; (**d**) $m=3,N_{3}=2$.

**Figure 11:**
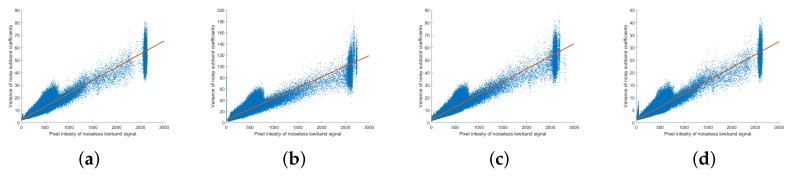
Noise analysis of real image in the NSP and NSCT domains after thresholding. (**a**) variance of noise subband coefficients ($\mathrm{Var}(L_{m})$) against pixel intensity of mean low-band image (${\bar{G}}_{m}$) at the scale level m=3; (**b**–**d**) variance of noise subband coefficients ($\mathrm{Var}(L_{m,n})$) against pixel intensity of mean low-band image (${\bar{G}}_{m}$) at the scale level *m* and direction level n=0 for (**b**) $m=1,N_{1}=8$; (**c**) $m=2,N_{2}=4$; (**d**) $m=3,N_{3}=2$.

We then analyzed low-dose X-ray noise using an actually obtained image. As shown in Figure 3, the low-dose X-ray images corresponding to the actual image have Poisson–Gaussian noise. Unlike the noise-generated images, saturation does not occur in the actual image near the maximum pixel value. Figure 10 shows real noisy image noise analysis in the NSP and NSCT domains. Since the noise tendencies in the NSP and NSCT domains are all similar, only one scale level is indicated for the NSP domain and only one direction level for each scale level in the NSCT domain. Unlike the previously simulated images, a single main noise distribution and several branches appear at the coarse level. In addition to noise generated during the attenuation process, actual X-ray images also exhibit edge changes as a result of photon scattering. As the branches outside of the main distribution are likely to cause errors in noise estimation, the noise should be analyzed separately. The detailed subband of the multiscale transform is sparse, with noise primarily included in the small values of the coefficients while edge information is primarily included in the large section. Using these properties, the noise can be analyzed by thresholding only small absolute values of the detailed subband coefficients. In this experiment, the thresholding value was set to 150 and the analysis limited to the section in which $|L_{m}|\leq 150$ and $|L_{m,n}|\leq 150$. The actual noise in the NSP and NSCT domains following thresholding is shown in Figure 11, from which it is seen that the real noise in the NSP and NSCT domain is Poisson–Gaussian distributed and follows Equations (5) and (6), respectively.

## 4. Noise Parameter Estimation

Next, noise parameters were obtained for each scale and direction level and the noise parameters were estimated by analyzing these noise parameters. The noise parameters $\alpha {}_{m,n}$ and $\sigma {}_{m,n}$ of Equation (6) were estimated using the noise distribution results obtained in the previous section. In the Var(L) and $\bar{G}$ graph of the simulated noisy image, the noise variance appears to deviate from that derived from Equation (6) as a result of saturation near the minimum and maximum pixel values; thus, for accurate estimation, the noise parameters were estimated within the range in which saturation does not occur using the following rule:

(7) $$\left({\bar{G}}_{m},\mathrm{Var}(L_{m,n})\right)=\left\{\left({\bar{G}}_{m},\mathrm{Var}(L_{m,n})\right)|10\leq \bar{G_{m}}\leq 220\right\}.$$

Using the rule in Equation (7), the mean of low-band image, ${\bar{G}}_{m}$, and the noise variance of subband coefficients, $L_{m,n}$, were used to produce *k* samples from which Equation (6) could be constructed:

(8) $$\mathrm{Var}(L_{m,n})\left(i\right)\approx \alpha {}_{m,n}{\bar{G}}_{m}\left(i\right)+\sigma {}_{m,n}^{2},\;i=1,2,\cdots ,k.$$

Equation (8) can be converted into a matrix equation using the selected *k* samples as follows:

(9) b=Ca,

where

(10) $$b=\left[\begin{array}{c} \mathrm{Var}(L_{m,n}\left(1\right))\\ \mathrm{Var}(L_{m,n}\left(2\right))\\ \vdots \\ \mathrm{Var}(L_{m,n}\left(k\right)) \end{array}\right],\;C=\left[\begin{array}{cc} {\bar{G}}_{m}\left(1\right) & 1\\ {\bar{G}}_{m}\left(2\right) & 1\\ \vdots & \vdots \\ {\bar{G}}_{m}\left(k\right) & 1 \end{array}\right],\;a=\left[\begin{array}{c} \alpha_{m,n}\\ \sigma_{m,n}^{2} \end{array}\right].$$

Using the least-square method, a can be estimated a using pseudo inverse matrix as

(11) $$a=\left(C^{T}C\right){}^{-1}C^{T}b.$$

The noise parameters of the full-band and each subband in the NSP and NSCT domains were estimated using Equation (11) and are listed in Table 1.

The estimated noise parameter values in the full-band are similar to those used in noise generation (α=0.0844 and $\sigma {}^{2}=32.5125$). This means that the noise estimation method using the least-squares method has very high accuracy. Moreover, as the NSP scale level coarsens, the Poisson and Gaussian noises both decrease. The Poisson noise ratios of the full-band and scale level subband are similar to the ratios of the Gaussian noise to the scale level. The rate at which the noise parameter decreases for each scale level is listed in Table 2.

The Poisson and Gaussian ratios are similar for each level in the full-band and to the NSP subband ratios, i.e., the Poisson and Gaussian noise decrease at the same rate for each scale and follow a ratio related to the NSP decomposition.

**Figure 12:**
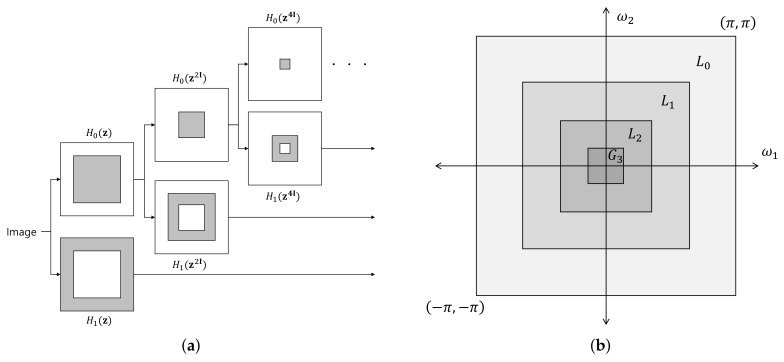
Non-subsampled pyramid decomposition. (**a**) decomposition scheme; (**b**) bandwidth of subbands.

Figure 12 shows a schematic of NSP decomposition and the bandwidth of the decomposed subbands. If the low- and high-pass filters of NSP are denoted by $H_{0}\left(z\right)$ and $H_{1}\left(z\right)$, respectively, $\Psi_{m}\left(z\right)$—the NSP subband decomposing filter of *m*-th scale level ($L_{m}$)—can be expressed as follows:

(12) $$\Psi_{m}\left(z\right)=\left\{\begin{array}{cc} H_{1}\left(z\right), & \mathrm{if}\;m=1,\\ H_{1}\left(z^{2^{\left(m-1\right)}I}\right)\cdot \prod_{k=1}^{m-1},H_{0}\left(z^{2^{\left(k-1\right)}I}\right), & \mathrm{if}\;m>1. \end{array}\right.$$

**Figure 13:**
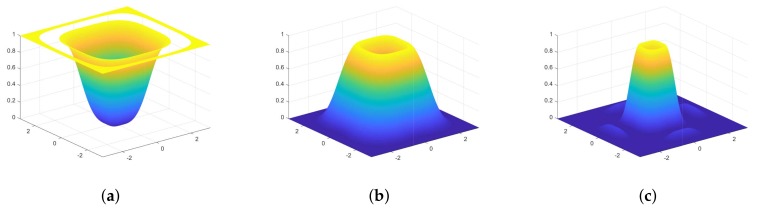
The magnitude of $\left|\Psi_{m}\left(z\right)\right|$ for (**a**) m=1; (**b**) m=2; (**c**) m=3.

The magnitude of $\Psi_{m}\left(z\right)$ is shown in Figure 13. For m=1 and m≥1, $\Psi_{m}\left(z\right)$ is, respectively, a high- and band-pass filter, and the passband of $\Psi_{m}\left(z\right)$ becomes narrower as the scale level becomes coarser. The energy of $\Psi_{m}\left(z\right)$, $E_{m}$, can be calculated as follows:

(13) $$E_{m}=\frac{1}{\left(j2\pi \right){}^{2}}\oint_{C}\left|\Psi_{m}\left(z\right)\right|{}^{2}dz.$$

The calculated values of $E_{m}$—$E_{1}=0.769155$, $E_{2}=0.178771$, and $E_{3}=0.044181$—are similar to the noise parameter ratios shown in Table 2, indicating that the Poisson–Gaussian noise in the NSP is reduced at the same rate as the energy of the decomposition filter for each scale. The relationship between the Poisson Gaussian noise of the full-band and NSP domain is given as

(14) $$\alpha {}_{m}=\alpha \cdot E_{m},\;\sigma {}_{m}^{2}=\sigma {}^{2}\cdot E_{m}.$$

We then estimated the noise parameters after passing through the NSDFB. As shown in Table 1, NSDFB distributes the noise uniformly to each directional subbands, which can be expressed as

(15) $$\alpha {}_{m,n}=\alpha {}_{m}/N_{m}=\frac{\alpha \cdot E_{m}}{N_{m}},\;\sigma {}_{m,n}^{2}=\sigma {}_{m}^{2}/N_{m}=\frac{\sigma {}^{2}\cdot E_{m}}{N_{m}}.$$

The Poisson–Gaussian noise parameters in the NSCT domain were then estimated as follows. First, the noise in the full-band was reduced in accordance with the energy ratio of the NSP decomposition filter as it passes through the NSP. Second, NSDFB divided the noise in the NSP domain equally by the number of directional decomposition levels.

Finally, the noise value of the actual low-dose X-ray image was obtained. Unlike the simulated noisy image, this image had no saturation but had a distribution of real noise that included several branches rather than one main distribution over an image range of 12 bits. Therefore, a candidate group different from that obtained using Equation (7) had to be set using the threshold method applied in the previous section. A sample group for the analysis of the actual noise image was obtained as follows:

(16) $$\left({\bar{G}}_{m},\mathrm{Var}(L_{m,n})\right)=\left\{\left({\bar{G}}_{m},\mathrm{Var}(L_{m,n})\right)|\;|L_{m,n}|\leq 150\right\}.$$

Table 3 shows the estimated noise parameters of the low-dose X-ray image obtained from Equation (11). The noise ratio of the full-band and the NSP subband decrease and converge with the energy ratio of the NSP decomposition filter, while the noise ratios of the NSP and NSCT subbands are reduced proportionally to the number of decomposition direction levels of NSDFB. Thus, the ratios of actual noise in both the NSP and NSCT domains also satisfy Equations (14) and (15).

## 5. Experimental Method and Results

To investigate the effects of the proposed method for accurate noise estimation on noise reduction performance, we compared its performance with that of an existing noise reduction technique. The thresholding method is a widely used noise reduction method that applies multiscale transforms such as wavelet, curvelet, and NSCT. The thresholding method can be broadly divided into hard and soft thresholding, and the threshold value, which is determined by the noise intensity, has a large effect on the noise reduction performance. The relationship between the noise level and the variances of a noisy and original image is obtained from Equation (1) as

(17) $$\sigma {}_{y}^{2}=\sigma {}_{x}^{2}+\sigma {}_{\eta }^{2}.$$

The variance of an observed image can be divided into the variance of its corresponding noiseless image and the variance of the noise. The most popular noise estimation method in the wavelet domain—the robust median estimator in multiscale transform proposed by Donoho in [28]—involves the use of the median of the absolute deviation of the subband coefficients. This estimator is useful for sparse signals such as wavelet, curvelet, and contourlet transforms because the subbands of the finest level have almost no signal power and are composed instead of mostly noise power. Applying the robust median estimator in [28] to the NSCT, the estimated noise standard deviation, $\sigma {}_{RM}$, is derived as follows:

(18) $$\sigma {}_{RM}\approx \frac{\mathrm{Median}\left(|L_{m,n}|\right)}{0.6745}.$$

Du et al. proposed Poisson noise reduction using a thresholding method in wavelet transform in [29]. In this method, the threshold is determined adaptively using the robust median estimator and the variance of the subband coefficient. Applying Du’s method to the NSCT domain, $\sigma {}_{y}^{2}$ can be estimated as follows:

(19) $$\sigma {}_{y}^{2}=E^{2}\left[L_{m,n}\right]=E\left[L_{m,n}^{2}\right]-\left(E\left[L_{m,n}\right]\right){}^{2}=E\left[L_{m,n}^{2}\right].$$

Because $L_{m,n}$ has no DC component, $E\left[L_{m,n}\right]=0$. The variance of an observed signal can be calculated as the average of the squares of the subband coefficients. From Equations (17)–(19), the standard deviation of an unknown noiseless signal can be estimated as

(20) $$\sigma {}_{x}=\sqrt{\max\left(\sigma {}_{y}^{2}-\sigma_{RM}^{2},0\right)}.$$

Because the standard deviation, $\sigma {}_{x}$, cannot have a negative value or be obtained directly, it is assumed to be zero if a negative number is found in the estimation process. The threshold, *T*, is determined to minimize Bayesian risk $r\left(T\right)=E\left(\hat{x}-x\right){}^{2}=E_{x}E_{y|x}\left(\hat{x}-x\right){}^{2}$ where $\hat{x}$ is the Bayesian estimate of the *x*. The adaptive threshold *T* is then obtained as

(21) $$T=\frac{\sigma {}_{RM}^{2}}{\sigma {}_{x}}.$$

The hard and soft thresholding methods are both processed using the value of *T* obtained by Equation (21).

In the proposed method, it is possible to calculate $\sigma {}_{\eta }$ using the estimated parameters without estimating $\sigma {}_{RM}$ for each subband by taking the expected value in Equation (1),

(22) $$E\left[y\right]=E\left[x+\sigma {}_{\eta }\left(x\right)\delta \right]=E\left[x\right]+E\left[\sigma {}_{\eta }\left(x\right)\right]E\left[\delta \right]=E\left[x\right],$$

because $E\left[\delta \right]=0$. The expected value of Equation (2) is then expressed as

(23) $$E\left[\sigma {}_{\eta }^{2}\left(x\right)\right]=E\left[\alpha x\right]+E\left[\sigma {}^{2}\right]=\alpha E\left[x\right]+\sigma {}^{2}.$$

As $E\left[x\right]=E\left[y\right]$ from Equation (22) and $\sigma {}_{RM}^{2}=E\left[\sigma_{\eta }^{2}\right]$, we can rewrite Equation (23) as

(24) $$\sigma {}_{RM}^{2}=E\left[\sigma {}_{\eta }^{2}\left(x\right)\right]=\alpha E\left[y\right]+\sigma {}^{2}.$$

Using $E\left[y\right]$ and the estimated noise parameters, *T* can be easily calculated.

Du et al.’s method [29] estimates the noise parameter for each subband at each time, resulting in inevitable sorting owing to the median calculation used in the process. Sorting takes a long time to execute, with the execution time increasing with the number of objects sorted. In addition, regardless of the type of noise, only one threshold value necessary for removing noise, $\sigma {}_{RM}$, can be obtained. By contrast, the proposed method is able to estimate the noise parameters of each subband using the estimated full-band noise parameters at once using Equation (15). Furthermore, it can estimate the parameters of Poisson and Gaussian noise, respectively.

**Figure 14:**
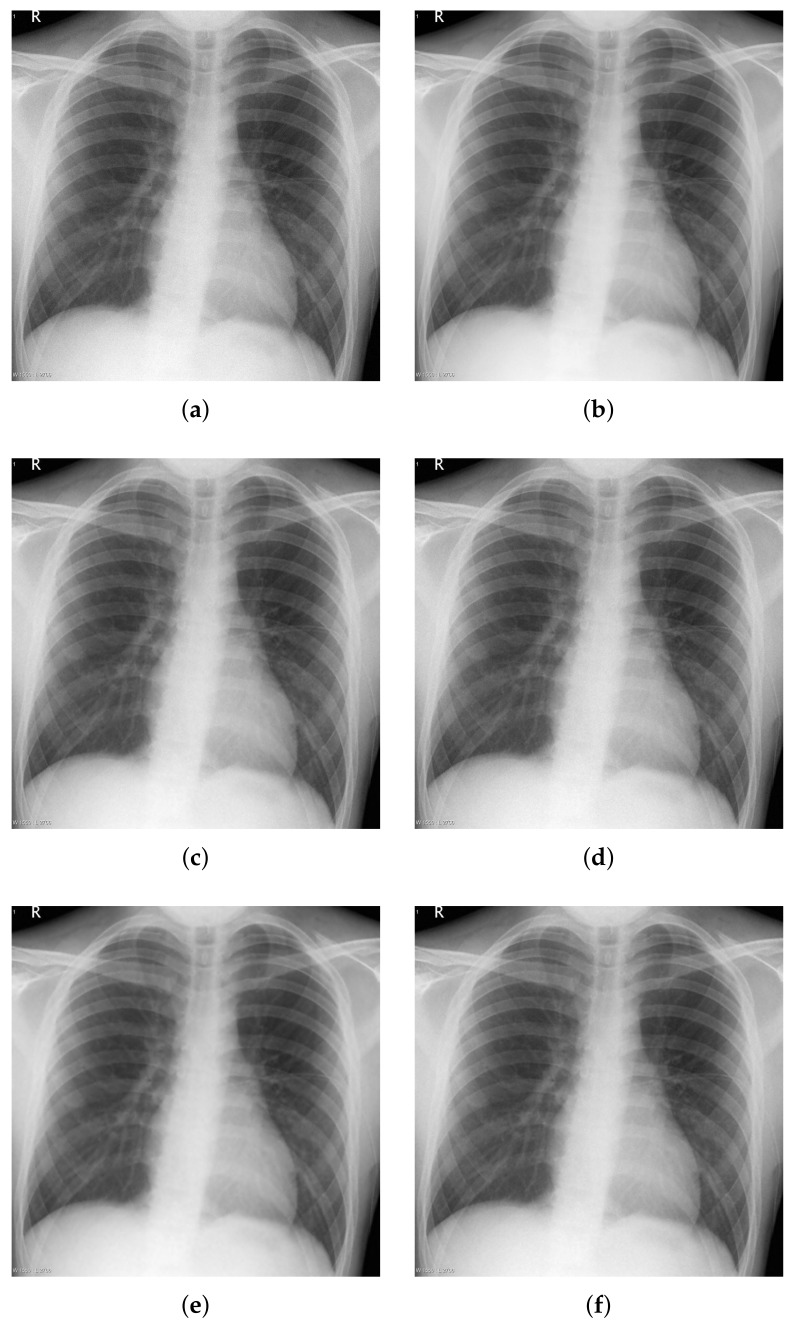
Denoising experiments for Poisson–Gaussian-corrupted image. (**a**) image with Poisson–Gaussian noise; (**b**) original image; (**c**–**f**) denoising results; (**c**) Du’s method [29] + hard threshold; (**d**) proposed method + hard threshold; (**e**) Du’s method [29] + soft threshold; (**f**) proposed method + soft threshold.

**Figure 15:**
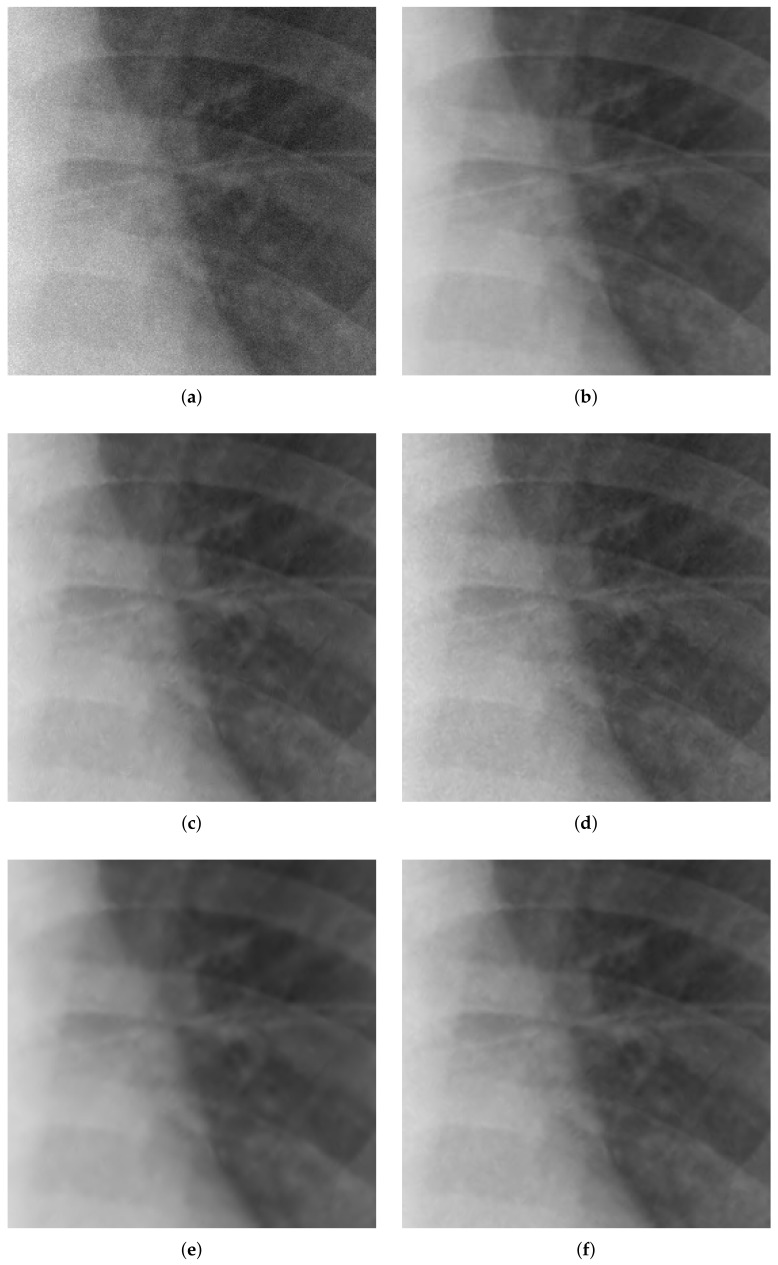
Partially magnification of Figure 14. (**a**) image with Poisson–Gaussian noise; (**b**) original image; (**c**–**f**) denoising results; (**c**) Du’s method [29] + hard threshold; (**d**) proposed method + hard threshold; (**e**) Du’s method [29] + soft threshold; (**f**) proposed method + soft threshold.

Noise reduction under both hard and soft thresholding was then performed using the threshold value, *T*, obtained from Du et al.’s method [29] and from the proposed method, and the results were compared. Figure 14 shows the noise reduction results for a Poisson–Gaussian corrupted image, with Figure 15 providing a partial magnified view of Figure 14. It is seen that noise was eliminated using both the hard and soft threshold methods. While the hard threshold method produced a small artifact, the results of the soft method were much smoother overall. Furthermore, the noise removal results obtained using the threshold value obtained from Du’s method were smoother than those obtained using the threshold value of the proposed method. Table 4 provides a quantitative evaluation and comparison of the noise reduction results in terms of mean squared error (MSE), peak signal-to-noise ratio (PSNR), and structural similarity (SSIM). The similarity in terms of noise removal results indicates that the thresholds obtained by the respective methods are similar, which in turn suggests that the proposed method performs well in terms of noise analysis and estimation.

**Figure 16:**
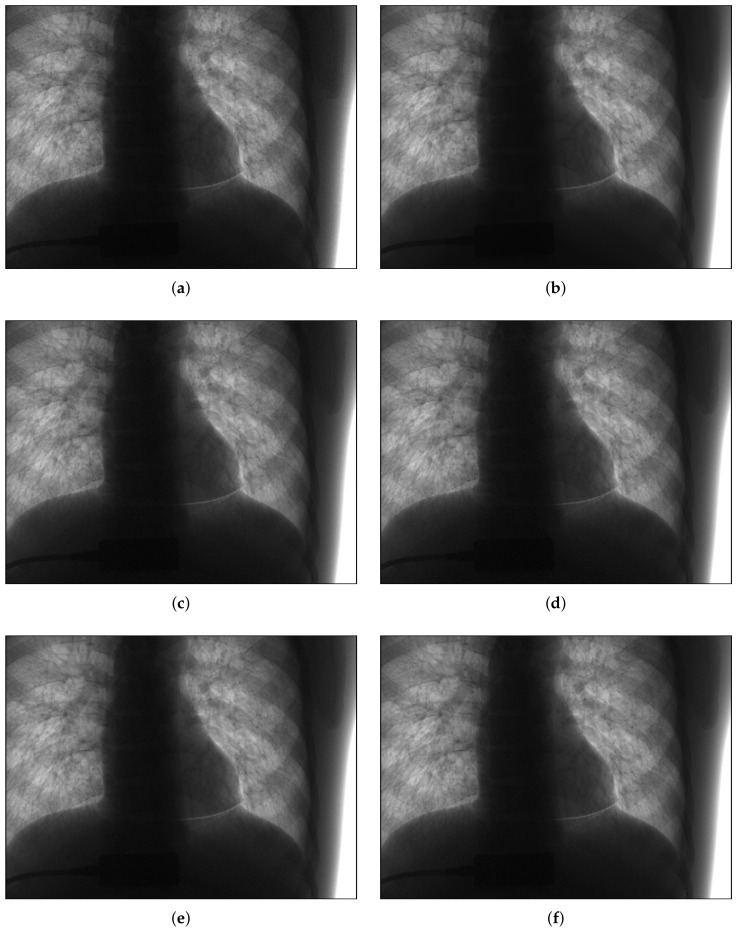
Denoising experiments for real noisy image. (**a**) low-dose X-ray noisy image; (**b**) noiseless image; (**c**–**f**) denoising results; (**c**) Du’s method [29] + hard threshold; (**d**) proposed method + hard threshold; (**e**) Du’s method [29] + soft threshold; (**f**) proposed method + soft threshold.

**Figure 17:**
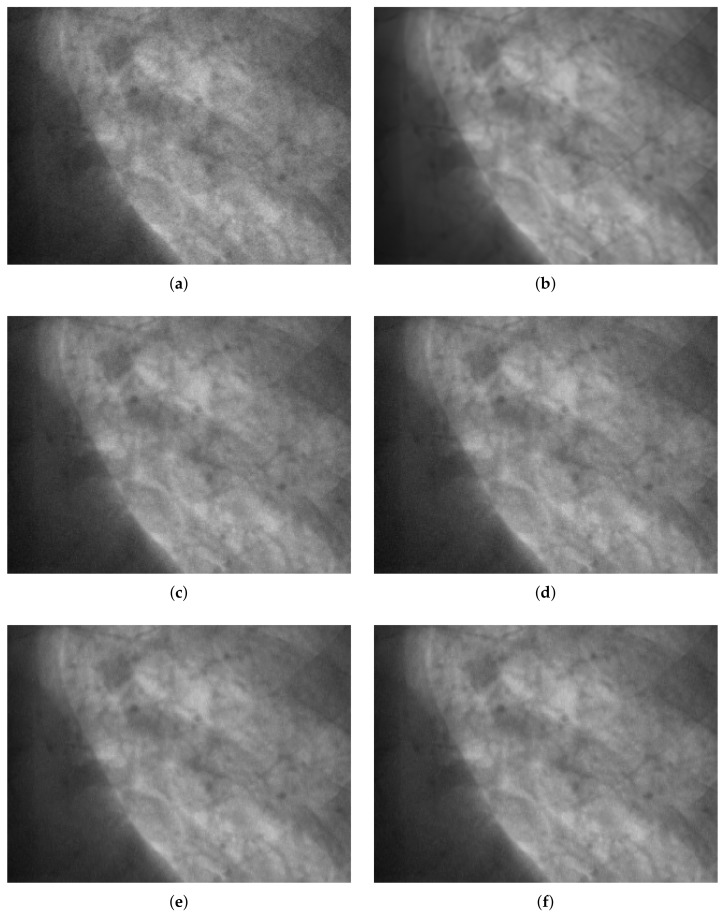
Partially magnification of Figure 16. (**a**) low-dose X-ray noisy image; (**b**) noiseless image; (**c**)–(**f**) denoising results; (**c**) Du’s method [29] + hard threshold; (**d**) proposed method + hard threshold; (**e**) Du’s method [29] + soft threshold; (**f**) proposed method + soft threshold.

Noise reduction was then performed on real low-dose X-ray images with Poisson–Gaussian noise. Figure 16 shows the noise reduction results obtained using the threshold values from Du’s method and the proposed method under the application of hard and soft thresholding methods (a magnification is shown in Figure 17), and Table 5 provides a numerical analysis of the noise results by threshold method and threshold-setting method. The results obtained using Du’s method and the proposed method were similar in both quantitative and qualitative cases for hard and soft thresholding. Since the noise reduction method uses the same threshold method, there is no significant improvement in the noise reduction performance. The only factor affecting noise reduction performance is the threshold value; hence, the similarity of the two sets of results suggests that the thresholds of Du’s method and the proposed method are similar, which implies that the noise parameter estimation of the proposed method has high accuracy.

## 6. Conclusions

In this paper, the Poisson–Gaussian noise of low-dose X-ray images in the NSCT domain has been analyzed. X-ray image sensor noise comprises Poisson noise originating from X-ray photons and Gaussian noise generated in the sensor. In a preliminary theoretical analysis, Poisson and Poisson–Gaussian noise were analytically generated and assessed. Noise in the NSCT domain has been analyzed by first analyzing the noise in the NSP domain and then passing the results to the NSCT domain for stepwise analysis. The results reveal that the noise in the NSCT subband also has a Poisson–Gaussian distribution comprising multiscale, low-band dependent Poisson noise and signal-independent Gaussian noise. Subsequent analysis of actual low-dose X-ray noise confirmed the consistency of the real noise in the NSCT domain with the theoretical analysis results. The noise parameters in the NSCT subband were then estimated using these noise analysis results. To do this, the Poisson–Gaussian noise was divided by the ratio of the energy of each decomposition filter in the NSP detail layer, with the noise of the NSP detail layer evenly distributed among the NSDFB direction levels as it passed through this filter. Using the proposed and an existing noise estimation method with both hard and soft thresholding techniques, the Poisson–Gaussian noise was removed in the multiscale domain, and the results based on qualitative and quantitative evaluation were found to be similar. The similarity of the noise removal results indicates that the proposed noise analysis and estimation method is accurate and has been properly developed. In the multiscale transform domain, the conventional noise estimation method can robustly obtain only median values, while the proposed method can quickly obtain both Poisson and Gaussian noise parameters. Based on our analysis findings that the subband noise depends on the low-band signal, we believe that our proposed method will be applicable to other noise elimination methods, including noise reduction methods based on multiscale transform.

## Figures and Tables

**Table 1:** Estimated noise parameters for simulated noisy image in NSP and NSCT domains.

*m*	*n*	$\alpha {}_{m,n}$	$\sigma {}_{m,n}^{2}$
Full-band		0.085417	32.533870
0	NSP	0.065490	25.032060
1		0.015346	5.813242
2		0.003845	1.433520
0	0	0.008101	3.113986
	1	0.007989	3.066360
	2	0.008038	3.076296
	3	0.008290	3.192081
	4	0.008321	3.190195
	5	0.008037	3.071675
	6	0.008069	3.087203
	7	0.008573	3.265702
1	0	0.003774	1.424735
	1	0.003838	1.444526
	2	0.003820	1.466331
	3	0.003909	1.483386
2	0	0.001915	0.709337
	1	0.001930	0.725090

**Table 2:** Ratio of NSP subband noise parameters those in full-band.

*m*	$\alpha {}_{m}/\alpha$	$\sigma {}_{m}^{2}/\sigma {}^{2}$
0	0.766709	0.769415
1	0.179659	0.178682
2	0.045014	0.044062

**Table 3:** Estimated noise parameters for real low-dose X-ray image in NSP and NSCT domains.

*m*	*n*	$\alpha {}_{m,n}$	$\sigma {}_{m,n}^{2}$
Full-band		0.466315	51.910244
0	NSP	0.357528	39.940532
1		0.083778	9.275465
2		0.020991	2.287289
0	0	0.037924	5.083407
	1	0.047112	5.444466
	2	0.047549	5.320386
	3	0.038998	4.907889
	4	0.040472	4.923046
	5	0.051170	4.922164
	6	0.051729	4.797308
	7	0.041266	4.814809
1	0	0.020238	2.351813
	1	0.020622	2.310953
	2	0.021323	2.317017
	3	0.021655	2.293817
2	0	0.010413	1.126114
	1	0.010583	1.161471

**Table 4:** Quantitative evaluation of denoising results of simulated Poisson–Gaussian noisy image.

	MSE	PSNR	SSIM
Du’s method [29] + Hard threshold	12.86810	37.03574	0.929491
Proposed method + Hard threshold	10.96603	37.73034	0.950617
Du’s method [29] + Soft threshold	10.60109	37.87733	0.960309
Proposed method + Soft threshold	10.32063	37.99378	0.961132

**Table 5:** Quantitative evaluation of low-dose X-ray image denoising results.

	MSE	PSNR	SSIM
Du’s method [29] + Hard threshold	2650.051	38.01466	0.966992
Proposed method + Hard threshold	2641.592	38.02854	0.967035
Du’s method [29] + Soft threshold	1627.141	40.13295	0.973222
Proposed method + Soft threshold	159.0478	40.23192	0.973426

## References

[1] Cesarelli M., Bifulco P., Cerciello T., Romano M., Paura L. (2013). X-ray fluoroscopy noise modeling for filter design. Int. J. Comput. Assist. Radiol. Surg..

[2] Elbakri I.A., Fessler J.A. (2002). Statistical image reconstruction for polyenergetic X-ray computed tomography. IEEE Trans. Med. Imaging.

[3] Anscombe F.J. (1948). The transformation of Poisson, binomial and negative-binomial data. Biometrika.

[4] Makitalo M., Foi A. (2013). Optimal inversion of the generalized Anscombe transformation for Poisson-Gaussian noise. IEEE Trans. Image Process..

[5] Buades A., Coll B., Morel J.M. (2005). A review of image denoising algorithms, with a new one. Multiscale Model. Simul..

[6] Kervrann C., Boulanger J. (2006). Optimal spatial adaptation for patch-based image denoising. IEEE Trans. Image Process..

[7] Aharon M., Elad M., Bruckstein A. (2006). *rmk*-SVD: An algorithm for designing overcomplete dictionaries for sparse representation. IEEE Trans. Signal Process..

[8] Hirakawa K., Parks T.W. (2006). Image denoising using total least squares. IEEE Trans. Image Process..

[9] Hammond D.K., Simoncelli E.P. (2008). Image modeling and denoising with orientation-adapted Gaussian scale mixtures. IEEE Trans. Image Process..

[10] Portilla J., Strela V., Wainwright M.J., Simoncelli E.P. (2003). Image denoising using scale mixtures of Gaussians in the wavelet domain. IEEE Trans. Image Process..

[11] Dabov K., Foi A., Katkovnik V., Egiazarian K. (2006). Image denoising with block-matching and 3 D filtering. Proc. SPIE.

[12] Le T., Chartrand R., Asaki T.J. (2007). A variational approach to reconstructing images corrupted by Poisson noise. J. Math. Imaging Vis..

[13] Bindilatti A.A., Mascarenhas N.D. (2013). A nonlocal poisson denoising algorithm based on stochastic distances. IEEE Signal Process. Lett..

[14] Salmon J., Harmany Z., Deledalle C.A., Willett R. (2014). Poisson noise reduction with non-local PCA. J. Math. Imaging Vis..

[15] Antonini M., Barlaud M., Mathieu P., Daubechies I. (1992). Image coding using wavelet transform. IEEE Trans. Image Process..

[16] Figueiredo M.A., Nowak R.D. (2003). An EM algorithm for wavelet-based image restoration. IEEE Trans. Image Process..

[17] Luisier F., Blu T., Unser M. (2011). Image denoising in mixed Poisson–Gaussian noise. IEEE Trans. Image Process..

[18] Do M.N., Vetterli M. (2005). The contourlet transform: An efficient directional multiresolution image representation. IEEE Trans. Image Process..

[19] Zhang X., Jing X. (2010). Image denoising in contourlet domain based on a normal inverse Gaussian prior. Digit. Signal Process..

[20] Do M.N., Vetterli M. (2003). The finite ridgelet transform for image representation. IEEE Trans. Image Process..

[21] Starck J.L., Candès E.J., Donoho D.L. (2002). The curvelet transform for image denoising. IEEE Trans. Image Process..

[22] Do M.N., Vetterli M. Contourlets: A directional multiresolution image representation. Proceedings of the 2002 International Conference on Image Processing.

[23] Po D.Y., Do M.N. (2006). Directional multiscale modeling of images using the contourlet transform. IEEE Trans. Image Process..

[24] Da Cunha A.L., Zhou J., Do M.N. (2006). The nonsubsampled contourlet transform: Theory, design, and applications. IEEE Trans. Image Process..

[25] Foi A., Trimeche M., Katkovnik V., Egiazarian K. (2008). Practical Poissonian-Gaussian noise modeling and fitting for single-image raw-data. IEEE Trans. Image Process..

[26] Do M.N., Vetterli M. (2003). Framing pyramids. IEEE Trans. Signal Process..

[27] Bamberger R.H., Smith M.J. (1992). A filter bank for the directional decomposition of images: Theory and design. IEEE Trans. Signal Process..

[28] Donoho D.L. (1995). De-noising by soft-thresholding. IEEE Trans. Inf. Theory.

[29] Du L., Wen Y., Ma J. Dual tree complex wavelet transform and Bayesian estimation based denoising of Poisson-corrupted X-ray Images. Proceedings of the 2013 IEEE Fourth International Conference on Intelligent Control and Information Processing (ICICIP).

